# Robust Iterative Distributed Minimum Total MSE Algorithm for Secure Communications in the Internet of Things Using Relays

**DOI:** 10.3390/s18113914

**Published:** 2018-11-13

**Authors:** Zhengmin Kong, Die Wang, Yunjuan Li, Chao Wang

**Affiliations:** 1 School of Electrical Engineering and Automation, Wuhan University, Wuhan 430072, China; zmkong@whu.edu.cn (Z.K.); wangdie1995@whu.edu.cn (D.W.); 2 School of Automation and Mechanical Engineering, Kunming University, Kunming 650118, China; 3 School of Information Engineering, Space Engineering University, Beijing 101400, China; drchaowang@126.com

**Keywords:** physical layer security, MIMO interference channel, relay, total MSE, IoT, imperfect channel estimation

## Abstract

In this article, we first investigate secure communications for a two-hop interference channel relay system with imperfect channel estimation in the wireless Internet of Things (IoT), where *K* source-destination pairs communicate simultaneously when an eavesdropper exists. We jointly conceive source, relay and destination matrices upon minimizing total mean-squared error (MSE) of all legitimate destinations while keeping the MSE at eavesdropper above a given threshold. We illuminate that the design of the source, relay and destination matrices is subject to both transmit power constraints and secrecy requirements. More specifically, we propose an efficient robust iterative distributed algorithm to simplify the process of the joint design for optimal source, relay and destination matrices. Furthermore, the convergence of the iterative distributed algorithm is described. Additionally, the performances of our proposed algorithm, such as its secrecy rate and MSE, are characterized in the form of simulation results. The simulation results reveal that the proposed algorithm is superior to the traditional approach. As a benefit, secure communications can be ensured by using the proposed algorithm for the multiple input multiple output (MIMO) interference relay IoT network in the presence of an eavesdropper.

## 1. Introduction

Future Internet of Things networks integrate the existing and evolving network with developments in communication and sensing fields, such as multi-hop, self-configuration and enhance the security of the communications with proper management to create an intelligent network that can be sensed [1,2,3,4,5,6]. Recently, with the rapid technological advancements of relay networks, wireless multi-hop relay networks (such as wireless sensor network) have become a popular technology for the future IoT networks [7,8,9,10,11]. Along with the enormous development in the field of wireless communication and hardware technology, wireless multi-hop relay networks are considered as major applications in IoT [12].

As the application scenarios in wireless IoT, the multi-hop relay networks consist of spatially distributed sensors or nodes, which enable IoT devices to collect and exchange data in relay manner. Since the broadcasting nature of wireless communications, this wireless IoT is more prone to eavesdropping [13]. Therefore, security is required, which can be accomplished by security approaches. Most of the security approaches for the wireless multi-hop IoT are deployed in the upper layers of the networks. However, nearly all upper-layer security approaches for IoT believe that the opponent or eavesdropper can obtain entirely control over a sensor or node by way of decoding cryptographic scheme [14]. Physical layer security technology, which comes from information theory to achieve perfect security [15,16,17], is found to be more robust than upper-layer security approaches for the IoT with multi-hop relay connectivity [13].

The physical layer security of the traditional close-range wireless systems mainly considers that the eavesdropper wiretaps the messages between sources and legitimate destinations. However, the security of long-range relays system considers that the eavesdropper wiretaps not only the messages between sources and relays but also the messages between relays and legitimate destinations. Therefore, the security of long-range relays system become more complexity than that of traditional wireless systems for close-range communication [18,19].

Physical layer security has been focused for multi-hop relay networks to combat eavesdropping for IoT [20,21,22,23,24,25]. In Reference [13], both channel aware encryption and precoding strategies are discussed in multi-hop IoT to achieve secrecy communication subject to resource constraints. In References [20,21], the authors select the optimal relay to improve security by joint relay and jammer selection algorithm, which may not fully take advantage of all relay nodes. In Reference [22], the problem of secure resource allocation for a two-way single relay wireless network is investigated, which is designed under schemes of applying and not applying cooperative jamming in the case of an eavesdropper. Security enhancement algorithm for IoT communication exposed to eavesdroppers has been forced on transmission design [23]. The authors in Reference [24] study the problem of improving security for the important data collection in IoT, where eavesdroppers can decode the signal extremely by combining their observations. The precoding matrices are optimized to satisfy that the MSEs at legitimate receivers are small and the MSE at eavesdropper is large in a relay aided cellular interference IoT system [25]. There are also some other schemes of achieving security, which are worth investigating. For instance, artificial noise has played an important role in enhancing the wireless communication physical layer secrecy in a two-hop relay network [26]. Furthermore, transmit beamforming is employed in an Amplify-and-Forward MIMO relay system, in order to obtain the maximum secrecy rate [27].

Although physical layer security for multi-hop relay IoT networks has been studied well, the resultant problem for secure communications still remains a significant challenge when the relay networks are faced with interferences and imperfect channel estimation. Some literature considers physical layer security problem just in interference channels. In Reference [28], a joint power control and beamforming algorithm is proposed for minimizing the total transmitted power, while keeping the signal-to-interference-plus-noise ratio (SINR) at each receiver over an expected threshold. An iterative distributed algorithm is used to design transmit precoding matrices and receive filter matrices for secure communications over the MIMO interference channels with an eavesdropper [29]. Other literatures just consider imperfect channel estimation. In Reference [30], an efficient beamforming approach has been proposed to combat eavesdropper with imperfect channel estimation. Secrecy outage analysis over a multiplicative composite channel model has been investigated with imperfect channel estimation [31].

To the best of our knowledge, the works on interference relay networks analysis and design for physical layer security in IoT system under imperfect channel estimation are still absent*.* Motivated by this challenge, we aim to provide secure communications for a two-hop relay system in future IoT with power supply strategy in this paper, where multiple source-destination pairs communicate simultaneously over the relay-interference channel in the presence of an active eavesdropper. In our article, we use MSE as the main performance metric. The system-wide minimum MSE (MMSE) has been considered in many works. In Reference [32], MMSE performance metric has been considered in a multiuser MIMO system where a distributed iterative algorithm and interference alignment are presented. In Reference [33], a weighted-MMSE method is proposed to apply in the optimization problems of sum-rate maximization, sum-MSE minimization and sum SINR maximization, respectively. However, the security problem of relays system is not considered in References [32,33].

To guarantee security in the relays system for IoT, we design an optimization scheme in order to minimize the total MSE estimated at legitimate destinations and keep the MSE at eavesdropper above an expected threshold, which is subject to the transmission power constrains at relay nodes and source nodes. To implement this optimization scheme, the source, relay and destination matrices must be jointly designed. Nevertheless, there exists a huge problem to design these matrices mentioned above, because the optimization scheme is too complicated to achieve the closed form solution or numerical solution of these matrices.

To conquer the above problem, we proposed an iterative distributed algorithm to simplify the optimization scheme. Specifically, for the sake of achieving the source, relay and destination matrices, we circularly calculate one of them by using the other two matrix variables obtained from previous iterations. Furthermore, Kronecker product is employed to facilitate the process of solving these matrix variables. Consequently, the acquisition of the numerical solution of the source, relay and destination matrices are much easier. Additionally, our simulation results demonstrate that the proposed iterative distributed algorithm will converge to a constant after several iterations. We also reveal that our proposed algorithm is superior to the traditional approach.

The remainder of the article is organized as follows. In Section 2, we describe the system model and propose the optimization problem. In Section 3, we propose an iterative distributed algorithm for dividing the non-convex optimization problem into three sub-problems. In Section 4, we demonstrate the convergence of the proposed algorithm. In Section 5, the simulation results are presented. In Section 6, the conclusions are summarized.

*Notation*: In this article, we use (.)*^H^* to represent Hermitian transpose, Tr(.) to represent the trace of a matrix, E{.} to represent the expectation, **I** to represent the identity matrix, **0** to represent a matrix or vector whose all element are zeros, $Y\sim CN\left(\mu ,\;\sigma^{2}\right)$ to represent Y following the complex normal distribution with mean μ and variance $\sigma^{2}$. bd(.) to represent a block-diagonal matrix, vec(.) to represent stack columns of a matrix on top of each other into a single vector, ‖.‖ to represent 2-norm of a vector, ⨂ to represent Kronecker product and ℂ to represent the complex field.

## 2. System Model and Methods

**Figure 1:**
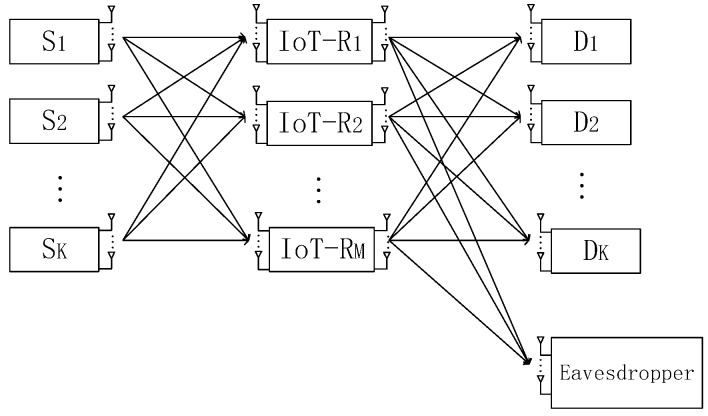
Two-hop multiple input multiple output (MIMO) interference relay system model in the wireless Internet of Things (IoT).

In this article, we investigate secrecy communication over the MIMO interference channels in two-hop relay system for the wireless IoT [34,35]. As shown in Figure 1, *K* source nodes transmit data to corresponding destination nodes by employing *M* IoT relay nodes. Meanwhile, an eavesdropper tries to wiretap the data from source. Assuming that the sophisticated eavesdropper can calculate its optimal receive matrix relying on minimizing its own total MSE [36]. Considering the path loss and transmission power constrains, the direct links between sources and destinations are negligible. According to previous related study [18,19], we assume that the eavesdropper is near by the relay and far away from the source. Therefore, the eavesdropper wiretapping the messages from both the sources and the relays simultaneously is difficult. Hence, we only consider the links from relay to eavesdropper and ignore the links from source to eavesdropper. The interference channels exist in the system when one of the source nodes transmits signal to corresponding destination while the others source nodes transmit signals synchronously.

In the system model, the sets of source nodes, relay nodes, corresponding destination nodes and the source-destination pairs are denoted as $\left\{S_{k}\right\}$, $\left\{R_{m}\right\}$, $\left\{D_{k}\right\}$ and $\left\{\left(S_{k},D_{k}\right)\right\}$, where k=1,…,K, m=1,…,M. More generally, the eavesdropper is denoted as *E*. Furthermore, $S_{k}$, $R_{m}$, $D_{k}$ and eavesdropper are equipped with $T_{k}$, $Q_{m}$, $N_{k}$ and $N_{e}$ antennas. We consider that the channels undergo slow varying flat Rayleigh fading. We also assume that the noise at all receiving nodes is additive white Gaussian noise (AWGN) with zero mean and variance $\sigma^{2}$ [37]. We denote $H_{km}$, $G_{mk}$ and $G_{me}$ as the actual channel matrices of $S_{k}-R_{m}$, $R_{m}-D_{k}$ and $R_{m}-E$ links.

The relays work in half-duplex model with amplify and forward (AF) strategy. So there needs two time slots to complete the data exchange between source and destination. In the first time slot, $S_{k}$ transmits data $s_{k}$ to $R_{m}$, then the $R_{m}$ receives the incoming signal with its receiving antennas and transmits $y_{r_{m}}$ to $D_{k}$ and eavesdropper in the second time slot. The received signals at $R_{m}$, $D_{k}$ and eavesdropper can be denoted as follows

(1) $$y_{r_{m}}=\sum_{k=1}^{K}H_{km}s_{k}+n_{r_{m}},m=1,\ldots ,M,$$

(2) $$y_{d_{k}}=\sum_{m=1}^{M}G_{mk}y_{r_{m}}+n_{d_{k}},k=1,\ldots ,K,$$

(3) $$y_{e}=\sum_{m=1}^{M}G_{me}y_{r_{m}}+n_{e},$$

where $y_{r_{m}}\in {\mathbb{C}}^{Q_{m}\times 1}$ is the received signal vector at relay $R_{m}$; $y_{d_{k}}\in {\mathbb{C}}^{N_{k}\times 1}$ is the received signal vector at destination $D_{k}$; $y_{e}\in {\mathbb{C}}^{N_{e}\times 1}$ is the received signal vector at the eavesdropper *E*; $H_{km}\in {\mathbb{C}}^{Q_{m}\times T_{k}}$ is denoted as channel gain between source $S_{k}$ and relay $R_{m}$; $G_{mk}\in {\mathbb{C}}^{N_{k}\times Q_{m}}$ is denoted as channel gain between relay $R_{m}$ and destination $D_{k}$; $G_{me}\in {\mathbb{C}}^{N_{e}\times Q_{m}}$ is denoted as channel gain between relay $R_{m}$ and eavesdropper *E*; $s_{k}\in {\mathbb{C}}^{T_{k}\times 1}$ is the transmitted data vector at source $S_{k}$; $n_{r_{m}}\in {\mathbb{C}}^{Q_{m}\times 1}$, $n_{d_{k}}\in {\mathbb{C}}^{N_{k}\times 1}$ and $n_{e}\in {\mathbb{C}}^{N_{e}\times 1}$ are AWGN vectors at $R_{m}$, $D_{k}$ and eavesdropper with zero mean and covariance matrix $\sigma_{r_{m}}^{2}I_{Q_{m}}$, $\sigma_{d_{k}}^{2}I_{N_{k}}$ and $\sigma_{e}^{2}I_{N_{e}}$.

To minimize total MSE at destinations and achieve secure communication, we jointly design transmit precoding matrices at source and relay and linear receive matrices at destinations and eavesdropper, which are subject to transmission power constrains at source and relay. For the sake of seeking optimum solution about above matrices, we scheme an iterative distributed algorithm.

Before transmitting the data $s_{k}$, we utilize transmit precoding matrix $U_{k}$ to encode the data $s_{k}$ at source $S_{k}$. Similarly, we utilize transmit precoding matrix $V_{m}$ to encode the data $y_{r_{m}}$ at relay $R_{m}$. The received signals at $R_{m}$, $D_{k}$ and eavesdropper are as follows

(4) $$y_{r_{m}}=\sum_{k=1}^{K}H_{km}U_{k}s_{k}+n_{r_{m}},m=1,\ldots ,M,$$

(5) $$y_{d_{k}}=\sum_{m=1}^{M}G_{mk}V_{m}y_{r_{m}}+n_{d_{k}},k=1,\ldots ,K,$$

(6) $$y_{e}=\sum_{m=1}^{M}G_{me}V_{m}y_{r_{m}}+n_{e},$$

In most scenarios, perfect channel estimation is considered. However, channel estimation is far from being perfect in realistic practical system. Hence, we assume imperfect channel estimation in our article. Here, $P_{sk}$ and $P_{rm}$ denote the maximum transmission power at $S_{k}$ and $R_{m}$. $N_{p}$ denotes the number of channel estimation pilot symbols. Considering ${\hat{H}}_{km}$, ${\hat{G}}_{mk}$ and ${\hat{G}}_{me}$ as the estimated channel matrices and $E_{h,km}$, $E_{r,mk}$, and $E_{e,me}$ as the MMSE estimation error matrices, respectively, we obtain the relationship of the estimated and actual channel matrices as $H_{km}={\hat{H}}_{km}+E_{h,km}$, $G_{mk}={\hat{G}}_{mk}+E_{r,mk}$, $G_{me}={\hat{G}}_{me}+\;E_{e,me}$, where $E_{h,km}\in {\mathbb{C}}^{Q_{m}\times T_{k}}\sim CN\left(0,\;{\left(1+\rho_{h}{\mathrm{SNR}}_{h}\right)}^{-1}\right)$ with $\rho_{h}=N_{p}/T_{k}$ and ${\mathrm{SNR}}_{h}=P_{sk}/\sigma_{r_{m}}^{2}$, $E_{r,mk}\in {\mathbb{C}}^{N_{k}\times Q_{m}}\sim CN\left(0,\;{\left(1+\rho_{r}{\mathrm{SNR}}_{r}\right)}^{-1}\right)$ with $\rho_{r}=N_{p}/Q_{m}$ and ${\mathrm{SNR}}_{r}=P_{rm}/\sigma_{d_{k}}^{2}$, and $E_{e,me}\in {\mathbb{C}}^{N_{e}\times Q_{m}}\sim CN\left(0,\;{\left(1+\rho_{e}{\mathrm{SNR}}_{e}\right)}^{-1}\right)$ with $\rho_{e}=N_{p}/Q_{m}$ and ${\mathrm{SNR}}_{e}=P_{rm}/\sigma_{e}^{2}$.

Finally, both destination $D_{k}$ and the eavesdropper employ linear receive matrix $W_{k}$ and $W_{e,k}$ respectively to receive the transmitted signals. Assuming imperfect channel estimation above mention, the estimate of the data $s_{k}$ at $D_{k}$ and the eavesdropper can be denoted as follows

(7) $${\hat{s}}_{k}=W_{k}^{H}y_{d_{k}}=W_{k}^{H}(\sum_{m=1}^{M}\sum_{l=1}^{K}\left(G_{mk}-E_{r,mk}\right)V_{m}\left(H_{lm}-E_{h,lm}\right)U_{l}s_{l}+\sum_{m=1}^{M}\;\left(G_{mk}-E_{r,mk}\right)V_{m}n_{r_{m}}+n_{d_{k}}),$$

(8) $${\hat{s}}_{ek}=W_{e,k}^{H}y_{e}=W_{e,k}^{H}(\sum_{m=1}^{M}\sum_{l=1}^{K}\left(G_{me}-E_{e,me}\right)V_{m}\left(H_{lm}-E_{h,lm}\right)U_{l}s_{l}+\sum_{m=1}^{M}\;\left(G_{me}-E_{e,me}\right)V_{m}n_{r_{m}}+n_{e}),$$

where $W_{k}$ and $W_{e,k}$ are the $T_{k}\times N_{k}$ and $T_{k}\times N_{e,k}$ receive weight matrices. We assume that $E\left\{s_{k}s_{k}^{H}\right\}=I_{T_{k}}$ is the covariance matrix of the data $s_{k}$ at $S_{k}$. From Equation (7), the MSE of estimating $s_{k}$ at $D_{k}$ can be calculated as

(9) $${\mathrm{MSE}}_{k}=tr\left(E\left\{\left({\hat{s}}_{k}-s_{k}\right){\left({\hat{s}}_{k}-s_{k}\right)}^{H}\right\}\right)=tr(\left(\sum_{m=1}^{M}W_{k}^{H}\left(G_{mk}-E_{r,mk}\right)V_{m}\left(H_{km}-E_{h,km}\right)U_{k}-I_{T_{k}}\right)\;(\sum_{m=1}^{M}W_{k}^{H}\left(G_{mk}-E_{r,mk}\right)V_{m}\left(H_{km}-E_{h,km}\right)U_{k}-I_{T_{k}}{)}^{H}+\sum_{m=1}^{M}\sigma_{r_{m}}^{2}W_{k}^{H}\left(G_{mk}-E_{r,mk}\right)V_{m}V_{m}^{H}{\left(G_{mk}-E_{r,mk}\right)}^{H}W_{k}+\;\sum_{m=1}^{M}\sum_{l=1,l\neq k}^{K}W_{k}^{H}\left(G_{mk}-E_{r,mk}\right)V_{m}\left(H_{lm}-E_{h,lm}\right)U_{l}U_{l}^{H}{\left(H_{lm}-E_{h,lm}\right)}^{H}V_{m}^{H}{\left(G_{mk}-E_{r,mk}\right)}^{H}W_{k}+\sigma_{d_{k}}^{2}W_{k}^{H}W_{k}).$$

Similarly, we can get the MSE of estimating $s_{k}$ at eavesdropper as follows

(10) $${\mathrm{MSE}}_{e,k}=tr\left(E\left\{\left({\hat{s}}_{ek}-s_{k}\right){\left({\hat{s}}_{ek}-s_{k}\right)}^{H}\right\}\right)=tr\;(\left(\sum_{m=1}^{M}W_{e,k}^{H}\left(G_{me}-E_{e,me}\right)V_{m}\left(H_{km}-E_{h,km}\right)U_{k}-I_{T_{k}}\right)\;{\left(\sum_{m=1}^{M}W_{e,k}^{H}\left(G_{me}-E_{e,me}\right)V_{m}\left(H_{km}-E_{h,km}\right)U_{k}-I_{T_{k}}\right)}^{H}+\sum_{m=1}^{M}\sigma_{r_{m}}^{2}W_{e,k}^{H}\left(G_{me}-E_{e,me}\right)V_{m}V_{m}^{H}{\left(G_{me}-E_{e,me}\right)}^{H}W_{e,k}+\;\sum_{m=1}^{M}\sum_{l=1,l\neq k}^{K}W_{e,k}^{H}\left(G_{me}-E_{e,me}\right)V_{m}\left(H_{lm}-E_{h,lm}\right)U_{l}U_{l}^{H}{\left(H_{lm}-E_{h,lm}\right)}^{H}V_{m}^{H}{\left(G_{me}-E_{e,me}\right)}^{H}W_{e,k}+\sigma_{e}^{2}W_{e,k}^{H}W_{e,k}).$$

The transmission power constraints at source $S_{k}$ and relay $R_{m}$ are as follows

(11) $$tr\left(U_{k}E\left\{s_{k}s_{k}^{H}\right\}U_{k}^{H}\right)\leq P_{sk},k=1,\ldots ,K,$$

(12) $$tr\left(V_{m}E\left\{y_{r_{m}}y_{r_{m}}^{H}\right\}V_{m}^{H}\right)\leq P_{rm},m=1,\ldots ,M,$$

where $P_{sk}$ and $P_{rm}$ denote the maximum transmission power at $S_{k}$ and $R_{m}$.

Without eavesdropper, the *K* legitimate communication pairs can achieve their maximum communication rates and the transmission is secure and reliable. However, when there exists an eavesdropper, the signals from source may be leaked out to the eavesdropper.

In order to elaborate more specifically and clearly, we assume a worst-case situation, namely in the presence of a sophisticated eavesdropper which can obtain the channel state information and our proposed algorithm, the eavesdropper calculates its linear receive matrix $W_{e,k}$ to minimize its own ${\mathrm{MSE}}_{e,k}$. To prevent this, we exploit the assumption to conceive the precoding/receive matrices for legitimate system at source or relay.

The solution of the source matrices $\left\{U_{k}\right\}$, relay matrices $\left\{V_{m}\right\}$ and destination matrices $\left\{W_{k}\right\}$ is vital. The solution of the problem is to utilize the source, relay and destination matrices to minimize the total MSE of all legitimate destination nodes and keep the ${\mathrm{MSE}}_{e,k}$ above an expected threshold $\varepsilon_{k}\;\left(k=1,\ldots ,K\right)$, while subjecting to the transmission power constrains at source and relay. The solution is denoted as follows

(13) $$\begin{array}{c} min\\ \left\{U_{k}\right\},\left\{V_{m}\right\},\left\{W_{k}\right\} \end{array}\;:\;\sum_{k=1}^{K}{\mathrm{MSE}}_{k}\;s.t.\;\;:\;{\mathrm{MSE}}_{e,k}\geq \varepsilon_{k},\;tr\left(U_{k}E\left\{s_{k}s_{k}^{H}\right\}U_{k}^{H}\right)\leq P_{sk},\;tr\left(V_{m}E\left\{y_{r_{m}}y_{r_{m}}^{H}\right\}V_{m}^{H}\right)\leq P_{rm},$$

where $\left\{U_{k}\right\},\;\left\{V_{m}\right\}$ and $\left\{W_{k}\right\}$ are the solution obtained.

## 3. The Iterative Distributed Algorithm of Solving Source, Relay, Destination and Eavesdropper Matrices

Due to the non-convex problem (13) with matrix variables, so we are facing an uphill battle to obtain the optimum solution of the joint design matrices. To deal with the solution, we design an iterative distributed algorithm to jointly design the optimal solution of the source matrices $\left\{U_{k}\right\}$, relay matrices $\left\{V_{m}\right\}$ and destination matrices $\left\{W_{k}\right\}$. The whole solving process of the three matrix variables is divided into three steps. We circularly calculate one of them by using the other two matrix variables obtained from previous iterations, the non-convex problem (13) is transformed into three sub-problems in each step.

The objective function of (13) can be denoted by total MSE (TMSE) as follows

(14) $$\mathrm{TMSE}=\sum_{k=1}^{K}{\mathrm{MSE}}_{k}.$$

### 3.1. Solution of Destination Matrices $\left\{W_{k}\right\}$

In the first iteration of our proposed algorithm, we set the initial value of $\left\{U_{k}\right\}$ and $\left\{V_{m}\right\}$, then calculate the optimal solution of $\left\{W_{k}\right\}$. Thus, at the following iteration of the algorithm, we calculate the optimal $\left\{W_{k}\right\}$ by utilizing previously obtained $\left\{U_{k}\right\}$ and $\left\{V_{m}\right\}$.

It is obvious from (13) that $\left\{W_{k}\right\}$ are independent with transmission power constrains of source and relay. Therefore, we can obtain the optimal linear receive matrices $\left\{W_{k}\right\}$ to minimize the total MSE at destination by the well-known linear MMSE receiver [38], which can be formulated as

(15) $$W_{k}={\left(\sum_{m=1}^{M}\sum_{l=1}^{K}\left(G_{mk}-E_{r,mk}\right)V_{m}\left(H_{lm}-E_{h,lm}\right)U_{l}U_{l}^{H}{\left(H_{lm}-E_{h,lm}\right)}^{H}V_{m}^{H}{\left(G_{mk}-E_{r,mk}\right)}^{H}\;+\sum_{m=1}^{M}\sigma_{r_{m}}^{2}\left(G_{mk}-E_{r,mk}\right)V_{m}V_{m}^{H}{\left(G_{mk}-E_{r,mk}\right)}^{H}+\sigma_{d_{k}}^{2}I_{N_{k}}\right)}^{-1}\;\left(\sum_{m=1}^{M}\left(G_{mk}-E_{r,mk}\right)V_{m}\left(H_{km}-E_{h,km}\right)U_{k}\right).$$

Assuming that the eavesdropper employs the above well-known linear MMSE method to calculate its linear receive matrix $W_{e,k}$., which can be formulated as

(16) $$W_{e,k}={\left(\sum_{m=1}^{M}\sum_{l=1}^{K}\left(G_{me}-E_{e,me}\right)V_{m}\left(H_{lm}-E_{h,lm}\right)U_{l}U_{l}^{H}{\left(H_{lm}-E_{h,lm}\right)}^{H}V_{m}^{H}{\left(G_{me}-E_{e,me}\right)}^{H}\;+\sum_{m=1}^{M}\sigma_{r_{m}}^{2}\left(G_{me}-E_{e,me}\right)V_{m}V_{m}^{H}{\left(G_{me}-E_{e,me}\right)}^{H}+\sigma_{e}^{2}I_{N_{e}}\right)}^{-1}\;\left(\sum_{m=1}^{M}\left(G_{me}-E_{e,me}\right)V_{m}\left(H_{km}-E_{h,km}\right)U_{k}\right).$$

### 3.2. Solution of Source Matrices $\left\{U_{k}\right\}$

After obtaining the optimal matrices $\left\{W_{k}\right\}$, we can calculate the transmit precoding matrices $\left\{U_{k}\right\}$ with $\left\{W_{k}\right\}$ obtained from current iteration and $\left\{V_{m}\right\}$ obtained from the previous iteration.

For further analysis, the TMSE of (14) can be specifically written as

(17) $$\mathrm{TMSE}=\sum_{k=1}^{K}tr\left(\sum_{m=1}^{M}\sum_{l=1}^{K}W_{k}^{H}\left(G_{mk}-E_{r,mk}\right)V_{m}\left(H_{lm}-E_{h,lm}\right)U_{l}U_{l}^{H}{\left(H_{lm}-E_{h,lm}\right)}^{H}V_{m}^{H}{\left(G_{mk}-E_{r,mk}\right)}^{H}W_{k}\right)-\;\sum_{k=1}^{K}tr\left(\sum_{m=1}^{M}W_{k}^{H}\left(G_{mk}-E_{r,mk}\right)V_{m}\left(H_{km}-E_{h,km}\right)U_{k}\right)+\sum_{k=1}^{K}tr\left(\sum_{m=1}^{M}U_{k}^{H}{\left(H_{lm}-E_{h,lm}\right)}^{H}V_{m}^{H}{\left(G_{mk}-E_{r,mk}\right)}^{H}W_{k}\right)+\;\sum_{k=1}^{K}tr\left(\sum_{m=1}^{M}\sigma_{r_{m}}^{2}W_{k}^{H}\left(G_{mk}-E_{r,mk}\right)V_{m}V_{m}^{H}{\left(G_{mk}-E_{r,mk}\right)}^{H}W_{k}+\sigma_{d_{k}}^{2}W_{k}^{H}W_{k}+I_{T_{k}}\right)$$

Define $P_{k,m,l}=W_{k}^{H}\left(G_{mk}-E_{r,mk}\right)V_{m}\left(H_{lm}-E_{h,lm}\right)$, so (17) can be written as

(18) $$\mathrm{TMSE}=\sum_{k=1}^{K}tr\left(\sum_{m=1}^{M}\sum_{l=1}^{K}P_{k,m,l}U_{l}U_{l}^{H}P_{k,m,l}^{H}\right)-\sum_{k=1}^{K}tr\left(\sum_{m=1}^{M}P_{k,m,k}U_{k}\right)-\sum_{k=1}^{K}tr\left(\sum_{m=1}^{M}U_{k}^{H}P_{k,m,k}^{H}\right)\;+\sum_{k=1}^{K}tr\left(\sum_{m=1}^{M}\sigma_{r_{m}}^{2}W_{k}^{H}\left(G_{mk}-E_{r,mk}\right)V_{m}V_{m}^{H}{\left(G_{mk}-E_{r,mk}\right)}^{H}W_{k}+\sigma_{d_{k}}^{2}W_{k}^{H}W_{k}+I_{T_{k}}\right)$$

Define $P_{k,m}=\left[P_{k,m,1},\;P_{k,m,2},\;\ldots ,\;P_{k,m,K}\right]$, ${\hat{P}}_{k,k}=\sum_{m=1}^{M}P_{k,m,k}$, $U=bd\left(U_{1},\;U_{2},\;\ldots ,\;U_{K}\right)$.

(19) $$\mathrm{TMSE}=\sum_{k=1}^{K}tr\left(\sum_{m=1}^{M}P_{k,m}UU^{H}P_{k,m}^{H}\right)-\sum_{k=1}^{K}tr\left({\hat{P}}_{k,k}U_{k}\right)-\sum_{k=1}^{K}tr\left(U_{k}^{H}{\hat{P}}_{k,k}^{H}\right)+\gamma ,$$

where $\gamma =\sum_{k=1}^{K}tr\left(\sum_{m=1}^{M}\sigma_{r_{m}}^{2}W_{k}^{H}\left(G_{mk}-E_{r,mk}\right)V_{m}V_{m}^{H}{\left(G_{mk}-E_{r,mk}\right)}^{H}W_{k}+\sigma_{d_{k}}^{2}W_{k}^{H}W_{k}+I_{T_{k}}\right)$. γ is independent with $\left\{U_{k}\right\}$, so it can be ignored in the solving process. Let $\hat{P}=bd\left[{\hat{P}}_{1,1},\;{\hat{P}}_{2,2},\ldots ,\;{\hat{P}}_{K,K}\right]$, from (19) we can get

(20) $$\mathrm{TMSE}=\sum_{k=1}^{K}tr\left(\sum_{m=1}^{M}P_{k,m}UU^{H}P_{k,m}^{H}\right)-tr\left(\hat{P}U\right)-tr\left(U^{H}{\hat{P}}^{H}\right)+\gamma .$$

To solve the above problems simplistically, we introduce some important formulas of Reference [39]

$$\mathrm{tr}\left(A^{H}B\right)={\left(vec\left(A\right)\right)}^{H}vec\left(B\right),\;\;\mathrm{tr}\left(A^{H}BAC\right)={\left(vec\left(A\right)\right)}^{H}\left(C^{H}⨂B\right)vec\left(A\right),\;vec\left(ABC\right)=\left(C^{H}⨂A\right)vec\left(B\right).$$

And define u≜vec(U) and $U_{k}=t_{k}Ut_{k}^{H}$, where $t_{k}=\left[0_{T_{k}\times \overset{k-1}{\underset{l=1}{\sum }}T_{l}}\;I_{T_{k}\times T_{k}}\;0_{T_{k}\times \overset{K}{\underset{l=k+1}{\sum }}T_{l}}\right]$. We can further simplify Formula (20) as follows

(21) $$\mathrm{TMSE}=\;u^{H}\omega u-\psi u-u^{H}\psi^{H}+\gamma ,$$

where $\tau =bd\left(I_{T_{1}},\;I_{T_{2}},\;\ldots ,\;I_{T_{K}}\right)$, $\omega =\sum_{k=1}^{K}\sum_{m=1}^{M}\tau ⨂P_{k,m}^{H}P_{k,m}$, $\psi ={\left(vec\left({\hat{P}}^{H}\right)\right)}^{H}$.

In the same way, we can obtain the simplified formula of ${\mathrm{MSE}}_{e,k}$ as follows

(22) $${\mathrm{MSE}}_{e,k}=u^{H}\omega_{e,k}u-\psi_{e,k}u-u^{H}\psi_{e,k}^{H}+\gamma_{e},$$

where $P_{e,k,m,l}=W_{e,k}^{H}\left(G_{me}-E_{e,me}\right)V_{m}\left(H_{lm}-E_{h,lm}\right)$, $P_{e,k,m}=\left[P_{e,k,m,1},\;P_{e,k,m,2},\;\ldots ,\;P_{e,k,m,K}\right]$, ${\hat{P}}_{e,k,k}=\sum_{m=1}^{M}P_{e,k,m,k}$, $\omega_{e,k}=\sum_{m=1}^{M}\tau ⨂P_{e,k,m}^{H}P_{e,k,m}$, $\gamma_{e}=tr\left(\sum_{m=1}^{M}\sigma_{r_{m}}^{2}W_{e,k}^{H}\left(G_{me}-E_{e,me}\right)V_{m}V_{m}^{H}{\left(G_{me}-E_{e,me}\right)}^{H}W_{e,k}+\sigma_{e,k}^{2}W_{e,k}^{H}W_{e,k}+I_{T_{k}}\right)$ and $\psi_{e,k}={\left(vec\left({\hat{P}}_{e,k,k}^{H}\right)\right)}^{H}\left(t_{k}⊗t_{k}\right)$.

Because of $E\left\{s_{k}s_{k}^{H}\right\}=I_{T_{k}}$, the transmission power constrains (11) can be rewritten as

(23) $$tr\left(t_{k}Ut_{k}^{H}{\left(t_{k}Ut_{k}^{H}\right)}^{H}\right)\leq P_{sk},k=1,\ldots ,K,$$

Then we obtain (24)

(24) $$u^{H}\rho u\leq P_{sk},k=1,\ldots ,K,$$

where $\rho =\left(t_{k}^{H}t_{k}\right)⊗\left(t_{k}^{H}t_{k}\right)$.

From (21), (22) and (24), the source matrices optimization problem is denoted as

(25) $$\;\begin{array}{c} min\\ \left\{U_{k}\right\} \end{array}\;:\;\mathrm{TMSE}\;\;s.t.\;:\;\;{\mathrm{MSE}}_{e,k}\geq \varepsilon_{k}\;\;u^{H}\rho u\leq P_{sk}$$

The source matrices optimization problem (25) is a quadratic constrained quadratic programming (QCQP) problem [40]. Compared with the non-convex problem (13), the (25) will be solved by the CVX of MATLAB toolbox [41].

### 3.3. Solution of Relay Matrices $\left\{V_{m}\right\}$

Since $\left\{W_{k}\right\}$, $\left\{W_{e,k}\right\}$ and $\left\{U_{k}\right\}$ are already obtained, the TMSE can be rewritten as

(26) $$\mathrm{TMSE}=\sum_{k=1}^{K}tr\left(\sum_{m=1}^{M}\sum_{l=1}^{K}{\bar{G}}_{mk}V_{m}{\bar{H}}_{lm}{\bar{H}}_{lm}^{H}V_{m}^{H}{\bar{G}}_{mk}^{H}\right)-\sum_{k=1}^{K}tr\left(\sum_{m=1}^{M}{\bar{G}}_{mk}V_{m}{\bar{H}}_{km}\right)-\;\;\sum_{k=1}^{K}tr\left(\sum_{m=1}^{M}{\bar{H}}_{km}^{H}V_{m}^{H}{\bar{G}}_{mk}^{H}\right)+\sum_{k=1}^{K}tr\left(\sum_{m=1}^{M}\sigma_{r_{m}}^{2}{\bar{G}}_{mk}V_{m}V_{m}^{H}{\bar{G}}_{mk}^{H}+\sigma_{d_{k}}^{2}W_{k}^{H}W_{k}+I_{T_{k}}\right)$$

where ${\bar{G}}_{mk}=W_{k}^{H}\left(G_{mk}-E_{r,mk}\right)$, ${\bar{H}}_{km}=\left(H_{km}-E_{h,km}\right)U_{k}$. Define $V=bd\left(V_{1},\;V_{2},\;\ldots ,\;V_{M}\right)$,${\bar{G}}_{k}=bd\left[{\bar{G}}_{1k},{\bar{G}}_{2k},\ldots ,{\bar{G}}_{Mk}\right]$, $\eta =bd\left[\sigma_{r_{1}}^{2}I_{Q_{1}},\sigma_{r_{2}}^{2}I_{Q_{2}},\ldots ,\sigma_{r_{M}}^{2}I_{Q_{M}}\right]$, ${\bar{H}}_{k}=bd\left[{\bar{H}}_{k1},{\bar{H}}_{k2},\ldots ,{\bar{H}}_{kM}\right]$, $\beta =\sum_{k=1}^{K}\left(\sigma_{d_{k}}^{2}W_{k}^{H}W_{k}+I_{T_{k}}\right)$. Then the TMSE can be simplified as

(27) $$\mathrm{TMSE}=\sum_{k=1}^{K}tr\left({\bar{G}}_{k}V\left(\sum_{l=1}^{K}{\bar{H}}_{l}{\bar{H}}_{l}^{H}\right)V^{H}{\bar{G}}_{k}^{H}\right)-\sum_{k=1}^{K}tr\left({\bar{G}}_{k}V{\bar{H}}_{k}\right)-\sum_{k=1}^{K}tr\left({\bar{H}}_{k}^{H}V^{H}{\bar{G}}_{k}^{H}\right)+\;\sum_{k=1}^{K}tr\left({\bar{G}}_{k}V\eta V^{H}{\bar{G}}_{k}^{H}\right)+\beta .$$

Let us introduce v=vec(V), then we obtain the TMSE as

(28) $$\mathrm{TMSE}=v^{H}\Omega v-Ov-v^{H}O^{H}+v^{H}\mu v+\beta ,$$

where $\Omega =\sum_{k=1}^{K}\left(\left(\sum_{l=1}^{K}{\bar{H}}_{l}{\bar{H}}_{l}^{H}\right)⨂\left({\bar{G}}_{k}^{H}{\bar{G}}_{k}\right)\right)$, $O=\sum_{k=1}^{K}{\left(vec\left({\bar{G}}_{k}^{H}{\bar{H}}_{k}^{H}\right)\right)}^{H}$, $\mu =\sum_{k=1}^{K}\left(\eta ⨂\left({\bar{G}}_{k}^{H}{\bar{G}}_{k}\right)\right)$.

In the same way, we can obtain the simplified formula of ${\mathrm{MSE}}_{e,k}$ as follows

(29) $${\mathrm{MSE}}_{e,k}=v^{H}\Omega_{e,k}v-O_{e,k}v-v^{H}O_{e,k}^{H}+v^{H}\mu_{e,k}v+\beta_{e,k},$$

where ${\bar{G}}_{e,k,m}=W_{e,k}^{H}\left(G_{me}-E_{e,me}\right)$, $\Omega_{e,k}=\left(\sum_{l=1}^{K}{\bar{H}}_{l}{\bar{H}}_{l}^{H}\right)⨂\left({\bar{G}}_{e,k}^{H}{\bar{G}}_{e,k}\right)$, $O_{e,k}={\left(vec\left({\bar{G}}_{e,k}^{H}{\bar{H}}_{k}^{H}\right)\right)}^{H}$, $\mu_{e,k}=\eta ⨂\left({\bar{G}}_{e,k}^{H}{\bar{G}}_{e,k}\right)$, $\beta_{e,k}=\sigma_{e}^{2}W_{e,k}^{H}W_{e,k}+I_{T_{k}}$, ${\bar{G}}_{e,k}=bd\left[{\bar{G}}_{e,k,1},{\bar{G}}_{e,k,2},\ldots ,{\bar{G}}_{e,k,M}\right]$.

Because of $E\left\{y_{r_{m}}y_{r_{m}}^{H}\right\}=\sum_{k=1}^{K}\left(H_{km}-E_{h,km}\right)U_{k}U_{km}^{H}{\left(H_{km}-E_{h,km}\right)}^{H}+\sigma_{r_{m}}^{2}I_{Q_{m}}$ and $V_{m}=d_{m}Vd_{m}^{H}$, $d_{m}=\left[0_{Q_{m}\times \sum_{l=1}^{m-1}Q_{l}}\;I_{Q_{m}\times Q_{m}}\;0_{Q_{m}\times \sum_{l=m+1}^{M}Q_{l}}\right]$. The transmission power constrains at relay can be denoted as

(30) $$v^{H}\lambda v\leq P_{rm},m=1,\ldots ,M,$$

where $\lambda ={\left(d_{m}^{H}\left(\sum_{k=1}^{K}\left(H_{km}-E_{h,km}\right)U_{k}U_{km}^{H}{\left(H_{km}-E_{h,km}\right)}^{H}+\sigma_{r_{m}}^{2}I_{Q_{m}}\right)d_{m}\right)}^{H}⊗\left(d_{m}^{H}d_{m}\right)$.

From (28), (29) and (30), the relay matrices optimization problem is denoted as

(31) $$\begin{array}{c} min\\ \left\{V_{m}\right\} \end{array}\;:\mathrm{TMSE}\;\;s.t.:\;\;{\mathrm{MSE}}_{e,k}\geq \varepsilon_{k}\;\;v^{H}\lambda v\leq P_{rm},m=1,\ldots ,M,$$

The relay matrices optimization problem (31) is a QCQP problem [40]. Compared with the non-convex problem (13), the (31) will be solved by utilizing the CVX of MATLAB toolbox [41].

The solving process of optimization matrices $\left\{W_{k}\right\}$, $\left\{U_{k}\right\}$ and $\left\{V_{m}\right\}$ by employing iterative distributed algorithm is summarized in Table 1 and variable *n* denotes the *n*th iteration.

At last, we introduce the communication rate and secrecy rate in this system model. The communication rate at destinations and eavesdropper are as follows [42],

(32) $$\mathrm{com}D_{k}=\log_{2}\left(1+\sum_{m=1}^{M}\frac{\|W_{k}^{H}G_{mk}V_{m}{\|}^{2}}{\left\|W_{k}^{H}W_{k}\right\|}\right),k=1,\ldots ,K,$$

(33) $$\mathrm{com}E=\log_{2}\left(1+\sum_{m=1}^{M}\frac{\|W_{e,m}^{H}G_{me}V_{m}{\|}^{2}}{\left\|W_{e,m}^{H}W_{e,m}\right\|}\right),$$

The secrecy rate at each destination can be obtained [43].

(34) $$\mathrm{Rate}D_{k}=max\left(0,\mathrm{com}D_{k}\right)-max\left(0,\mathrm{com}E\right),k=1,\ldots ,K.$$

## 4. The Convergence of the Proposed Algorithm

In this part, the convergence of our proposed algorithm is proved [44]. Since the $\left\{U_{k}\right\}$ and $\left\{V_{m}\right\}$ are updated at each iteration by minimizing the TMSE, the TMSE is reduced gradually after each iteration. Furthermore, it is obvious that the TMSE has a lower limit which is at least greater than 0. This implies that the proposed algorithm is convergence. The convergence of the proposed algorithm can be proved exactly as follows. According to Section 3, we can obtain the objective function is

(35) $$\begin{array}{c} min\\ \left\{U_{k}\right\},\left\{V_{m}\right\},\left\{W_{k}\right\} \end{array}\;:\;\sum_{k=1}^{K}{\mathrm{MSE}}_{k}$$

The solution of $\left\{W_{k}\right\}$ can be ignored in the proof of convergence, because it is calculated by obtained $\left\{U_{k}\right\}$ and $\left\{V_{m}\right\}$ rather than utilize optimal scheme. For the obtained $\left\{V_{m}\right\}$, the optimal solution can be denoted as follows.

(36) $$\begin{array}{c} min\\ \left\{U_{k}\right\} \end{array}\;:\;\sum_{k=1}^{K}{\mathrm{MSE}}_{k}$$

Therefore, we can get $\sum_{k=1}^{K}{\mathrm{MSE}}_{k}\left(U_{k}\left(n+1\right),V_{m}\left(n\right)\right)\leq \sum_{k=1}^{K}{\mathrm{MSE}}_{k}\left(U_{k}\left(n\right),V_{m}\left(n\right)\right)$. 

Similarly, for the obtained $\left\{U_{k}\right\}$, the optimal solution can be denoted as follows.

(37) $$\begin{array}{c} min\\ \left\{V_{m}\right\} \end{array}\;:\;\sum_{k=1}^{K}{\mathrm{MSE}}_{k}$$

Hence, we can deduce $\sum_{k=1}^{K}{\mathrm{MSE}}_{k}\left(U_{k}\left(n+1\right),V_{m}\left(n+1\right)\right)\leq \sum_{k=1}^{K}{\mathrm{MSE}}_{k}\left(U_{k}\left(n+1\right),V_{m}\left(n\right)\right)$. Furthermore, we get $\sum_{k=1}^{K}{\mathrm{MSE}}_{k}\left(U_{k}\left(n+1\right),V_{m}\left(n+1\right)\right)\leq \sum_{k=1}^{K}{\mathrm{MSE}}_{k}\left(U_{k}\left(n\right),V_{m}\left(n\right)\right)$.

**Figure 2:**
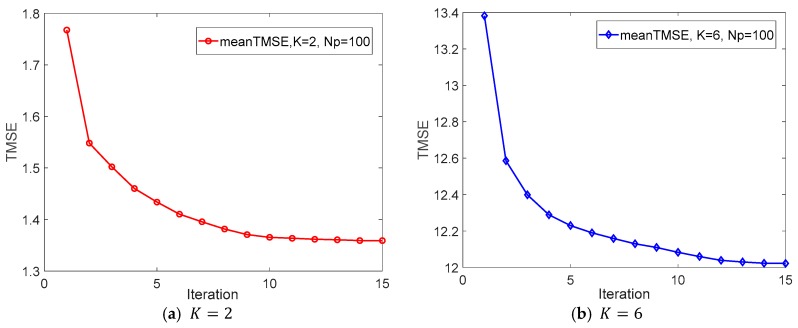
Total mean square error (TMSE) versus the number of iterations.

According to the mentioned above, we conclude that the TMSE is decreasing gradually with the updated $\left\{U_{k}\right\}$ and $\left\{V_{m}\right\}$ after each iteration. The TMSE converges to a constant after several iterations, which is also demonstrated by the Figure 2 of the Numerical Results part.

## 5. Numerical Results

In this part, we provide numerical results to examine the effectiveness of the optimization iterative distributed algorithm for secure transmission in interference channels MIMO relay system with eavesdropping. Assuming that all nodes have the same antennas, $T_{k}=Q_{m}=N_{k}=N_{e}=3$ and all channel matrices are independently distributed Gaussian channel matrices with zero mean and unit variance. The noises at all receiving nodes are assumed as AWGN with $\sigma_{d_{k}}^{2}=\sigma_{r_{m}}^{2}=\sigma_{e}^{2}=1$. The transmission power constrains at sources and relays are assumed as $P_{s_{k}}=P_{r_{m}}=20\;\mathrm{dB}$. Assuming that the eavesdropper knows the channel state information of the links between relay and itself. In addition, the threshold of eavesdropper’s MSE are $\varepsilon_{k}=2.2,\;k=1,\ldots ,K$. All figures are averaged over 1000 independent test. 

Figure 2 depicts the convergence of proposed iterative distributed algorithm, where we have K=2 or 6, M=3, $N_{p}=100$, as well as $P_{s_{k}}=P_{r_{m}}=20\;\mathrm{dB}$. As can be seen in Figure 2, TMSE decreases gradually until convergence when the number of iterations increases. It can be observed in both Figure 2a,b, as the system scale increases (i.e., increasing K), the convergence speed decreases and TMSE increases. This is because more legitimate source-destination pairs increase both the system complexity and the interferences between each legitimate source-destination pair and they lead to more iterations to approximate convergence and the increasement of TMSE.

**Figure 3:**
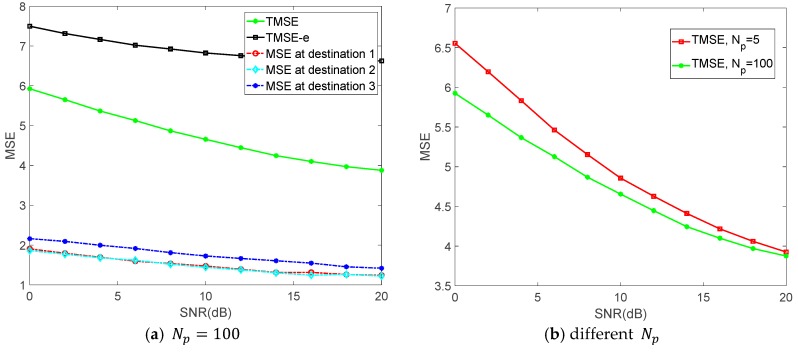
The TMSE and MSE versus signal to noise ratio (SNR).

Figure 3 shows the changes of TMSE and MSEs at different destinations versus signal to noise ratio (SNR) with different $N_{p}$, where we have K=3, M=3, TMSE-e denotes the $\sum_{k=1}^{K}{\mathrm{MSE}}_{e,k}$. As shown in Figure 3a, both TMSE of all destinations and MSE at different destinations decrease gradually as the SNR increases. It can also be observed in Figure 3a that MSEs at different destinations are very similar, statistically there is the nearly the same of the three legitimate links. Obviously, the TMSE of all legitimate destinations is much lower than the TMSE-e. It means that the system can be achieved a better transmission performance by employing our proposed algorithm against the eavesdropper. Additionally, Figure 3b shows that the TMSE of all destinations decreases when $N_{p}$ increases. The reason is that the reduction of $N_{p}$ causes the increasement of channel estimation errors, which in turn leads to the increasement of the TMSE.

**Figure 4:**
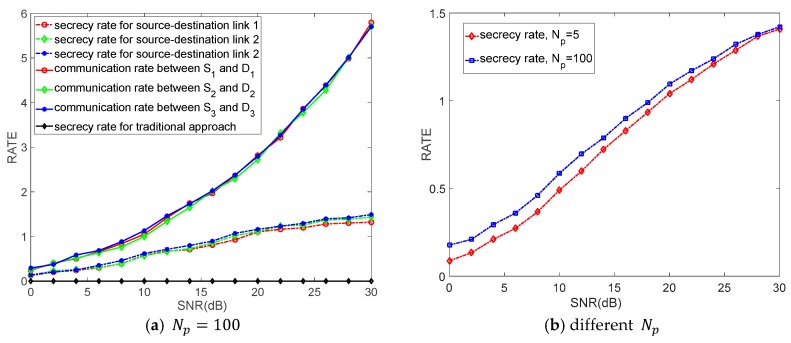
The communication rate and secrecy rate versus SNR.

Figure 4 depicts the values of secrecy rate and communication rate versus transmission power constrains with different $N_{p}$, when K=3, M=3, which is in the same background with Figure 3. As shown in Figure 4a, compared to the traditional approach, the proposed algorithm can support a positive secrecy rate and secrecy rate gradually improves with the SNR increasing. In other word, our proposed algorithm guarantees secure communications for the system. According to Reference [45], when the communication rate of legitimate transmitter-receiver link is lower than that of the transmitter-eavesdropper link, we define the secrecy rate as “0”; when the communication rate of legitimate transmitter-receiver link is larger than that of the transmitter-eavesdropper link, we define secrecy rate as the difference between the communication rate of legitimate transmitter-receiver link and that of the transmitter-eavesdropper link (as shown in (32)). The traditional approach does not consider the eavesdropper and the eavesdropper is so “sophisticated” (it knows our security algorithm) that the communication rate of legitimate transmitter-receiver link is lower than that of the transmitter-eavesdropper link. Consequently, no matter how large the SNR is, the secrecy rate is always zero. That communication rates of three links are similar, the situation of the secrecy rates is the same. Moreover, the achievable secrecy rates are lower than communication rates, because the proposed algorithm sacrifices a part of communication rate to realize a positive secrecy rate. Additionally, it can be observed in Figure 4b that the secrecy rate improves with the increasing of $N_{p}$. This is because the increasement of $N_{p}$ causes the reduction of channel estimation errors, which in turn leads to the increasement of secrecy rates.

**Figure 5:**
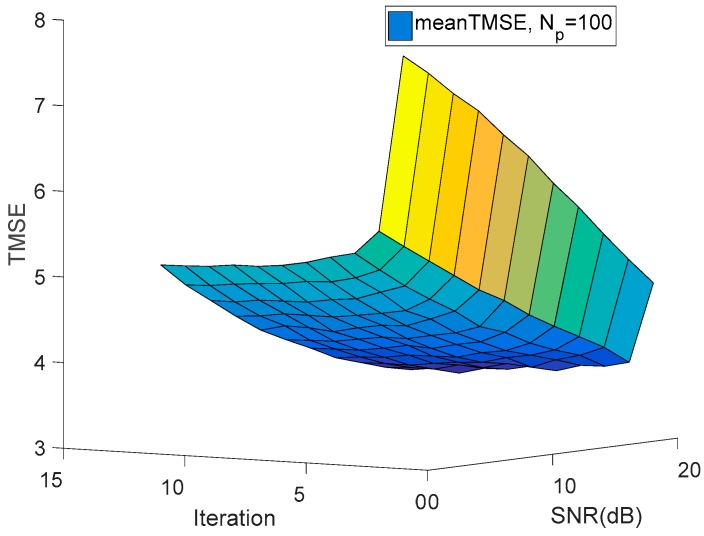
The TMSE versus the number of iterations and SNR.

Furthermore, in Figure 5 we depict the variation of the TMSE as a function of the number of iterations and the transmission power constrains, where we have K=3,M=3 and $N_{p}=100$ As shown in Figure 5, the TMSE of all destinations decreases when the SNR or the number of iterations increases. In addition, it also be depicted in Figure 5 that the TMSE decreases quickly at the beginning of the iteration process. And that means the proposed algorithm converges quickly.

## 6. Conclusions

In this article, we first investigate secure communication in MIMO interference relay IoT network in the presence of an active eavesdropper. A robust iterative distributed algorithm which jointly optimizes the source, relay and destination matrices has been proposed under the imperfect channel estimation. It aims to minimize the TMSE of all legitimate destinations subject to transmission power constrains while keeping MSE at eavesdropper above a certain threshold. The convergence of the proposed algorithm has also been proved. In addition, the performances of our proposed algorithm, such as its secrecy rate and MSE, are characterized in the form of simulation results. The simulation results reveal that our proposed algorithm is superior to the traditional approach. In other word, security can be ensured by using the proposed algorithm in the interference channel MIMO relay IoT network when there exists an eavesdropper.

## Figures and Tables

**Table 1:** The proposed iterative distributed algorithm for problem (13).

Steps	Specific Progress
Step 1	Set n=0, ${\mathrm{TMSE}}^{\left(n\right)}=0$ and initialize the $\left\{U_{k}^{\left(0\right)}\right\}$ and $\left\{V_{k}^{\left(0\right)}\right\}$ satisfying power constrains (11) and (12).
Step 2	Calculate $\left\{W_{k}^{\left(n+1\right)}\right\}$ and $\left\{W_{e,k}^{\left(n+1\right)}\right\}$ with $\left\{U_{k}^{\left(n\right)}\right\}$ and $\left\{V_{m}^{\left(n\right)}\right\}$ obtained from previous iteration.
Step 3	Update $\left\{U_{k}^{\left(n+1\right)}\right\}$ by solving the problem (25) with $\left\{W_{k}^{\left(n+1\right)}\right\}$, $\left\{W_{e,k}^{\left(n+1\right)}\right\}$ and $\left\{V_{m}^{\left(n\right)}\right\}$.
Step 4	Update $\left\{V_{m}^{\left(n+1\right)}\right\}$ by solving the problem (31) with $\left\{W_{k}^{\left(n+1\right)}\right\}$, $\left\{W_{e,k}^{\left(n+1\right)}\right\}$ and $\left\{U_{k}^{\left(n+1\right)}\right\},$ then calculate ${\mathrm{TMSE}}^{\left(n+1\right)}$.
Step 5	If ${\mathrm{TMSE}}^{\left(n+1\right)}-{\mathrm{TMSE}}^{\left(n\right)}\leq \xi$, then end; otherwise set n=n+1, then go to step 2.
